# Fear of Falling, Lower Extremity Strength, and Physical and Balance Performance in Older Adults with Diabetes Mellitus

**DOI:** 10.1155/2020/8573817

**Published:** 2020-06-03

**Authors:** Mantana Vongsirinavarat, Witaya Mathiyakom, Ratchanok Kraiwong, Vimonwan Hiengkaew

**Affiliations:** ^1^Faculty of Physical Therapy, Mahidol University, 999, Salaya, Phuttamonthon, Nakhon Pathom 73170, Thailand; ^2^Department of Physical Therapy, California State University, Northridge, 18111 Nordhoff Street, Jacaranda Hall 1513B, Northridge, CA 91330, USA

## Abstract

Fear of falling (FoF) is known to affect the physical activities and quality of life of older adults with type 2 diabetes mellitus (DM). Many complications of DM, especially ones distressing lower extremity (LE), could lead to increased fall risk and FoF. This study aimed to explore the relationship between FoF, LE muscle strength, and physical performance in older adults without diabetes mellitus (ONDM) and with DM (ODM) with varying degrees of balance impairment. The participants comprised 20 ONDM and 110 ODM. The ODM was grouped by the number of failed performances of the modified clinical test of sensory interaction and balance (mCTSIB). The scores of FoF, balance performance of mCTSIB, physical performance of TUG, and LE muscle strength were compared between groups. The results showed that FoF was present in 30% and 60% of the ONDM and ODM, respectively. Forty percent of the ODM failed one condition of the mCTSIB, while 18% and 16% failed two and three conditions, respectively. As the number of failed performances on the mCTSIB increased, the proportions of participants with FoF significantly increased. The psychosocial domain of FoF, LE muscle strength, and TUG score was significantly different between groups and more affected in the ODM with a greater number of failed performances on the mCTSIB. In conclusion, the mCTSIB can differentiate the varying degrees of balance impairment among ODM. FoF, LE muscle strength, and physical performance are more affected as the degree of balance impairment increases. Comprehensive management related to balance and falls in the ODM should include a regular evaluation and monitoring of standing balance, LE muscle strength, physical performance, and FoF.

## 1. Introduction

Recently, fear of falling (FoF) has been a significant concern of older adults due to its negative impact on the quality of life (QoL). The concept of FoF has evolved from a postfall syndrome to low perceived self-efficacy at avoiding falls during activities of daily living [1, 2]. The prevalence of FoF ranged from 33 to 46% in older adult nonfallers and was up to 85% in older adult fallers [3, 4]. This psychological symptom significantly decreases physical activity and independence, and increases fall risk and depression, all of which results in poor QoL [5]. Many factors such as older age, female gender, history of falling, cognitive impairment, frailty, anxiety, depression, dizziness, poor self-reported health perception, gait abnormalities, low economic status, and living alone have associated to the high prevalence of FoF [5, 6].

Diabetes mellitus (DM) is a significant risk factor for falls in older adults [7–10] and associated with an increase in FoF [11]. The prevalence of falls and FoF is significantly higher in older adults with DM (ODM) than in older adults without DM (ONDM) [8, 11–14]. Microvascular complications associated with DM result in multiple impairments including sensory deficits and muscle weakness due to peripheral neuropathy [15–18], loss of visual acuity due to retinopathy [18–20], and impaired postural control and falls due to vestibulopathy [21–23]. These impairments predispose ODM to an increase in fall risk [17, 20, 24], falls [12, 17, 25], and FoF [25].

Clinical balance assessment is essential to identify a patient's ability to maintain balance, fall risk, and potentially FoF in ODM. Currently, the sensory organization test (SOT) is considered the gold standard for identifying the ability to use different sensory inputs to maintain balance [26]. Unfortunately, the SOT is not readily available in many clinics. However, the modified clinical test of sensory interaction and balance (mCTSIB) [27] has been widely used in clinical practice. During the mCTSIB, patients were asked to maintain a static standing balance on a firm and compliant surface, with eyes open and closed to mimic the conditions of the SOT. The mCTSIB has been shown to have good test-retest reliability (*r* = 0.75) in older community-dwelling adults [28]. Small but significant correlations (*r* = 0.3-0.51) between the mCTSIB and SOT scores suggest its potential for identifying the varying degrees of impaired standing balance [29].

Since impaired sensation required for maintaining standing balance has been reported to vary largely among the ODM [17, 18, 20, 23, 24], it is plausible that ODM's performance on the mCTSIB may also vary among them and contribute to the degree of FoF they experienced. On the other hand, FoF may influence how well the ODM performs on the mCTSIB, particularly when they maintain standing balance on a compliant surface with their eyes closed. Unfortunately, to our knowledge, the relationship between ODM's ability to perform the mCTSIB and FoF has not been reported and needs further evaluation.

A decline in the motor system associated with DM may also influence FoF in ODM. The weakness of the lower extremity (LE) muscles is commonly observed among ODM [30, 31]. The ODM with FoF performed relatively poorer than the ONDM on many physical performance tests such as the five-time sit-to-stand test [30, 32], raising from a chair of knee height [4], Berg balance score [13], one-leg stance [13], and timed up and go (TUG) test [12, 13]. In fact, impaired performance on the TUG test is a good predictor for falls and FoF in older women with type 2 DM [32]. Although impaired physical performance in the ODM with FoF can be partially attributable to the weakness of the LE muscles in older adults [33, 34], the relationship between the LE strength and FoF, specifically in ODM, has not been reported. Additionally, weakness of the LE muscles may contribute to how well the ODM perform on the mCTSIB, physical performance tests, and the degree of FoF experienced by the ODM. The lack of understanding of the relationships between these measures warrants further investigation since it will allow clinicians to identify a comprehensive evaluation and management program for the ODM.

The aim of this study was to explore the relationships between FoF, LE muscle strength, physical performance, and balance in the ODM and ONDM. Primarily, it was hypothesized that FoF would be significantly greater in the ODM with a relatively greater degree of impairment in standing balance and LE muscle strength than those with a lower degree of impairments. Secondarily, LE muscle strength was hypothesized to decrease significantly as the degree of balance impairment increased.

## 2. Materials and Methods

### 2.1. Participants

The participants were recruited prospectively from the metabolic disease clinic in primary healthcare centres in Nakhon Pathom Province, Thailand. The ODM were included if they were of 60 years or older, with a diagnosis of type 2 DM for more than five years, able to walk independently at least ten meters and able to follow verbal instruction. The participants were excluded if they had a history of central nervous system conditions, amputation of LE, history of fracture, or surgery of lumbar and LE, having pain resulting in movement difficulty. Additionally, 20 ONDM were recruited from communities in the same province to serve as a control group. The older adults eligible to be included in the ONDM group were ones without a history of DM affirmed by a normal range of fasting blood sugar (FBS) and Hemoglobin A1C (HbA1C) levels. Additionally, they had no visual and vestibular impairments as well as no signs of peripheral neuropathy as confirmed by the clinical screening tests.

All participants signed an informed consent prior to the interview and testing. The study protocol was approved by the Mahidol University Central Institutional Review Board (MU-CIRB) (protocol no. 2015/035.0303).

### 2.2. Measurements

Personal and clinical characteristics including age, gender, and history of DM were recorded. FBS and HbA1C levels were documented.

The mCTSIB was used to identify participants' ability to maintain static standing balance [29]. This test is highly sensitive (88-91%) and moderately specific (50-57%) when using the SOT as a reference standard [29]. The participants were asked to stand with feet together for 30 seconds under four testing conditions: (1) eyes open and firm surface, (2) eyes closed and firm surface, (3) eyes open and foam surface, and (4) eyes closed and foam surface. The foam used in this study had medium density with a size of 24 inches of width and length and 4 inches of height (SunMate; Dynamic System Inc., Leicester, NC, USA). The participants performed three trials of each condition, and the average time was used to represent the condition. If the time was less than 30 seconds, the performance of that condition was considered failed. The sum of the average time of four conditions was used to represent the total mCTSIB score (mCTSIB-Tol).

The LE muscle torque at the midrange of knee flexors and extensors and ankle dorsiflexors and plantar flexors of the participants' dominant LE was measured using a hand-held dynamometer. After one practice trial, three trials were performed, and the data were averaged. The sum of the average of four muscle groups was used to represent the total LE muscle torque (LEMT-Tol).

The 34-item with six-point Likert scale from 1 (not at all) to 6 (very much) Thai Geriatric Fear of Falling Questionnaire [35] was used to assess the presence of FoF. This questionnaire has good (*r* = 0.87) test-retest reliability and good (*r* = 0.91) convergent validity against the Falls Efficacy Scale-International (FES-I) [35]. The maximum total FoF score (FoF-Tot) was 170 points. The 66-point cut-off was able to discriminate between participants with and without FoF with 90% sensitivity and 100% specificity [35]. Three subscales of physical (FoF-Phy), environmental (FoF-Env), and psychological (FoF-Psy) domains were also derived by using the scores of 15, eight, and 11 items from the questionnaire, respectively. The content validity of these domains was achieved by the consensus of four experienced physical therapists. Additionally, strong correlations between the score of each domain and the FES-I (*r* = −0.81 to -0.95) were observed [35].

The physical performance of the TUG test was used to determine a dynamic standing balance and gait. It has excellent test-retest reliability (ICC = 0.96-0.98) [36]. To perform this test, the participants sat in an armchair with back against the chair. They were then asked to stand up from a chair, walk three meters, turn around, walk back, and sit down with the back against the chair again as quickly and safely as possible. One practice trial was performed to familiarize with the task. The time used to complete a single trial was used for data analysis.

### 2.3. Data Analysis

The ODM were stratified into four groups based on the number of failed performances on the mCTSIB as ODM-0, ODM-1, ODM-2, and ODM-3. ODM-0 were the older adults with diabetes who successfully passed all 4 conditions of the mCTSIB. ODM-1 were the older adults with diabetes who failed one condition of the mCTSIB. ODM-2 were the older adults with diabetes who failed any two conditions of the mCTSIB. ODM-3 were the older adults with diabetes who failed more than two conditions of the mCTSIB. Using the FoF-Tol cut-off value of greater than 66 points, the participants were classified as with or without FoF. The proportion of participants with and without FoF relative to the number of failed performances was analyzed using a chi-square test. Between-group differences in the participant's characteristics, each LE muscle torque and its sum (LEMT-Tol) were tested using one-way analysis of variance (ANOVA). The LEMT-Tol was used as a covariate when identifying the differences in FoF-Tol and its domains between groups using one-way analysis of covariance (ANCOVA). Both LEMT-Tol and FoF-Psy were then used as covariates to identify the differences in mCTSIB-Tol and TUG using one-way ANCOVA. Post hoc analysis with Bonferroni's adjustment was used to control type I error for both ANOVA and ANCOVA. The level of significance was set at 0.05. All statistical analysis was performed using SPSS version 25.

## 3. Results

Twenty ONDM and 110 ODM participated in this study.  Table 1 summarizes the characteristics of participants in each group. All ONDM performed successfully on all conditions of the mCTSIB. Twenty-eight ODM also completed all conditions of the mCTSIB (ODM-0). Forty-four ODM failed condition 3 or 4 of the mCTSIB and were classified as ODM-1. Twenty and 18 ODM were ODM-2 and ODM-3, respectively. The ODM-2 and ODM-3 were significantly older (*p* < 0.05) than the ODM-0 and ONDM. FBS and HbA1C of the ODM groups were significantly higher (*p* < 0.05) than those of the ONDM. The duration since DM was diagnosed, FBS, and HbA1C were not significantly different among the ODM groups.

**Figure 1:**
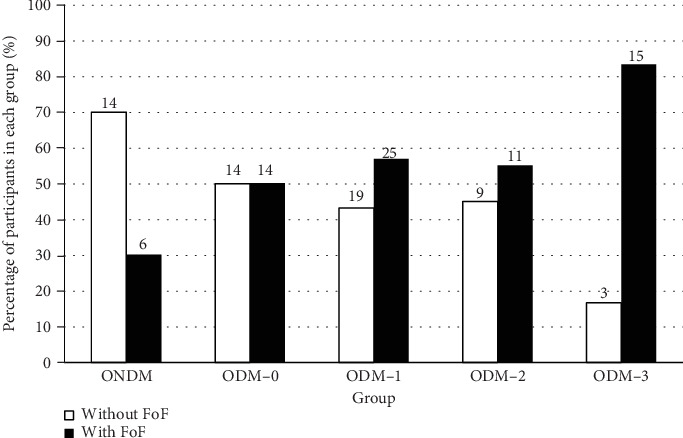
Proportion of participants with and without fear of falling (FoF). The number of each bar denoted the number of participants. ONDM = older adults without diabetes; ODM-0 = older adults with diabetes without failed performance on mCTSIB; ODM-1 = older adults with diabetes who failed on one condition of mCTSIB; ODM-2 = older adults with diabetes who failed on two conditions of mCTSIB; ODM-3 = older adults with diabetes who failed on more than two conditions of mCTSIB.

A significant association between FoF and the number of failed performances on the mCTSIB was observed (*p* = 0.024). The proportion of participants with FoF increased as the number of failed performances on the mCTSIB increased (Figure 1).

The LE muscle torque decreased as the number of failed performances on the mCTSIB increased and was significantly different between groups (Table 2). The knee extensor torque was significantly lower in the ODM-1 (*p* < 0.001), ODM-2 (*p* < 0.001), and ODM-3 (*p* < 0.001) than the ONDM and in the ODM-2 (*p* = 0.002) and ODM-3 (*p* = 0.014) than the ODM-0. The knee flexor and ankle plantar flexor torques were significantly lower in the ODM groups compared to the ONDM (*p* < 0.001). Likewise, the ODM-4 demonstrated a significantly lower knee flexor (*p* = 0.02) and ankle plantar flexor (*p* = 0.03) torques than the ODM-0. The ankle dorsiflexor torque was significantly lower in the ODM-2 (*p* = 0.004), ODM-3 (*p* < 0.001), and ODM-4 (*p* = 0.008) than the ONDM and in the ODM-3 (*p* = 0.033) than the ODM-1. As a result, the LEMT-Tol was significantly different between the ONDM and all ODM groups (*p* < 0.05), ODM-0 and ODM-2 (*p* = 0.01) and ODM-0 and ODM-3 (*p* = 0.02). Since the LE muscle torque might potentially play a role in the performance of the mCTSIB, TUG, and FoF, the LEMT-Tol was taken into account when comparing these outcomes between groups.

The FoF-Tol tended to increase as the number of failed performances on the mCTSIB increased (Table 2). ANCOVA with the LEMT-Tol as a covariate indicated no significant effect of the covariate on the FoF-Tol (*p* = 0.87), FoF-Phy (*p* = 0.46), FoF-Env (*p* = 0.87), and FoF-Psy (*p* = 0.34). A trend of the between-group difference in the FoF-Tol was observed (*p* = 0.09). No significant between-group difference in the FoF-Phy (*p* = 0.22) and FoF-Env (*p* = 0.39) was noted. In contrast, the FoF-Psy was significantly different between groups (*p* = 0.01). The FoF-Psy was significantly greater in the ODM-1 (*p* = 0.02), ODM-2 (*p* = 0.04), and ODM-3 (*p* = 0.03) than that of the ONDM. These results indicated that FoF-Psy was significantly greater in the ODM with impaired mCTSIB performance(s) as compared to the ONDM. Since between-group difference in FoF-Psy was observed, it was used as a covariate in addition to the LEMT-Tol when comparing the mCTSIB-Tol and TUG between groups.

Significant between-group differences in the mCTSIB-Tol were noted when the LEMT-Tot and FoF-Psy were taken into account (Table 2). The mCTSIB-Tol of the ONDM was significantly greater than that of the ODM-1 (*p* < 0.001), ODM-2 (*p* < 0.001), and ODM-3 (*p* < 0.001). No statistically significant effect of the LEMT-Tol (*p* = 0.48) and FoF-Psy (*p* = 0.52) as covariates on the mCTSIB-Tol was observed (Table 2).

There was a significant difference in the TUG score between groups (*p* = 0.05) when controlled for both covariates of the LEMT-Tol and FoF-Psy (Table 2). The TUG score was significantly longer in the ODM-3 (*p* = 0.04) as compared to that of the ONDM. However, no significant effect of the LEMT-Tol (*p* = 0.10) and FoF-Psy (*p* = 0.12) as covariates on the TUG score was observed (Table 2). The mean values of the TUG score of all ODM groups were greater than 11.1 seconds [37], suggesting an increase in fall risk in the ODM.

## 4. Discussion

This study aimed to explore the relationships between FoF and balance and physical performances and LE strength in older adults with and without type 2 DM. Our results demonstrated that FoF was observed in both the ONDM and ODM groups, and the proportion of participants with FoF increased as the number of failed performances on the mCTSIB increased. The mCTSIB-Tol differed between groups of ODM and were not significantly affected by the FoF-Psy and LEM-Tol. Additionally, the FoF-Psy, each LE muscle torque, and LEMT-Tol and TUG scores were significantly different between groups and more affected in the ODM with a greater number of failed performances on the mCTSIB. These results suggested that a static balance evaluation using the mCTSIB, a dynamic balance and gait using the TUG test, LE muscle strength, and the FoF should be regularly monitored in the ODM.

As expected, the proportion of older adults with FoF increased as the number of failed performances on the mCTSIB increased. Interestingly, the presence of FoF was also observed in the ONDM. The occurrence of FoF in the ONDM was within the lower range of 20.8-85% previously reported in the overall older adult population [6]. The presence of FoF in the ONDM signifies the needs to monitor FoF in all older adults.

Based on our study, the mCTSIB can sufficiently differentiate the degrees of balance impairment in the ODM. Poor postural control observed in the ODM is the result of sensory and motor deficits associated with microvascular complications of DM as well as FoF [11–13, 25]. A significant decrease in the mCTSIB-Tol score and the failed performances on the mCTSIB suggest an increase in the degrees of balance impairment. An increase in the number of failed performances on the mCTSIB indicates an increase in the number of sensory inputs that the ODM are unable to use to maintain balance. For example, a failed performance on condition 4 of the mCTSIB was used to identify vestibular impairment in patients with DM [21, 22]. Likewise, a failed performance on conditions 3 and 4 may indicate impaired use of visual and vestibular inputs to maintain balance. Although the mCTSIB-Tol score is moderately sensitive and specific relative to the composite score of the SOT [29], its ability to indicate an impaired use of specific sensory input to maintain balance needs further validation against the gold standard of SOT.

The declined muscle properties in ODM have been reported [30–32] even before the presenting of neuropathy [31]. Similar to our study, the lower LE strength observed in the ODM compared to that observed in the ONDM was reported in knee extensor [30, 31] and ankle dorsiflexor muscles [31]. A relatively poor muscle performance assessed by the sit to stand test [30, 32] as well as handgrip strength [30] was reported in the ODM as compared to the ONDM. Additionally, the lean muscle mass examined by muscle biopsy [30] and muscle volume of the lower extremity [31] were lower in the ODM as compared to those in the ONDM.

However, the decrease in LE strength and FoF do not interfere with the ODM's performance on the mCTSIB. The previous studies suggested that the performance on the mCTSIB was affected by LE strength [38] and possibly by FoF. Our study also indicated that as the numbers of failed performances on the mCTSIB increased, the LE muscle torques also decreased. The decrease in the LE muscle strength may interfere with the performance on the mCTSIB, particularly when the participants stood on a compliant surface during conditions 3 and 4. However, the between-group differences in the mCTSIB-Tol score were not significantly affected when the LEMT-Tol and FoF were taken into consideration. These different results could be attributable to the between-study differences in the degree of deficits in muscle strength [38]. These results suggest that the mCTSIB can be used to detect different degrees of balance impairment in the ODMs despite the deficits in LE muscle strength and FoF observed in the ODM.

A negative psychological impact of FoF on the ability to maintain balance is highlighted in our study. A significantly higher score of the FoF-Psy in the ODM with failed performances on the mCTSIB than the counterparts of ONDM and ODM-0 suggests a relatively higher psychological concerns related to FoF in the ODM. Significant between-group differences in the FoF-Phy and FoF-Env and a significantly greater FoF-Psy in all ODM with failed performances on the mCTSIB compared to the counterparts emphasize the psychological consequences of FoF and possibly the perceived functional restrictions in the ODM [13, 39]. Psychological attributes, especially depression, are reportedly associated with worse physical outcomes in ODM [32, 40]. Incorporating specific psychological outcomes, such as the Geriatric Depression Scale, into further research, may allow us to understand the relationship between different psychological attributes in ODM.

A decline in the performance of dynamic balance and gait is also observed as the number of failed performances on the mCTSIB increases and possibly leads to an increase in the risk of falling. The weakness of LE muscles as well as FoF may play a significant role in the TUG score [12, 13, 32]. However, no significant effects of FoF-Psy and LEMT-Tol as covariates on the TUG scores observed in our study suggest that these two variables have no significant influence on the ODM's performance of these tests. When controlled for these two variates, the TUG score of the ODM-3 was significantly lower than that of the ONDM. With the cut-off of 11.1 seconds for the TUG score [37], all ODM groups are considered to have risk of falling. These results underscore the clinical significance of the TUG test to detect fall risk in the ODM.

This study has several limitations. Firstly, it was conducted in a primary care setting where older adults were actively participating in health maintenance. Therefore, the sample of participants may bias toward the ODM who were likely to have fewer complications from DM and low prevalence for falling. The frailty is also unlikely in this study population. Therefore, a larger sample size in different settings will allow us to further understand the relationships between FoF, balance, LE strength, and physical performance of the ODM. Secondly, the sample size of ONDM as the control group was also rather small due to the stringent inclusion criteria. A larger number of ONDM may strengthen the statistical power and generalization of this study. Thirdly, other aspects of the psychological domain, such as depression and self-perceived health status, were not evaluated. Further studies on psychological issues, especially in ODM and their associations with FoF, physical attributes, and fall risk, would be constructive on the comprehensive fall prevention program. Other complications of diabetes, such as polyneuropathy and retinopathy, which may influence FoF and balance performance, were also not addressed. Lastly, due to the study design, the relationship between FoF and other variables of interest could neither determine causality nor identify the degrees of importance among them.

## 5. Conclusions

The presence of FoF is significantly associated with the ability to maintain balance based on the mCTSIB. As the ability to maintain balance declines, the FoF-Psy increases, while the lower extremity muscle strength and physical performance such as TUG decrease. The dynamic interactions among multiple systems of sensory impairment, motor and physical performance deficit, and psychosocial decline should be intensively monitored in ODM. Comprehensive management related to balance and falls in ODM should include regular monitoring of standing balance, LE muscle strength, physical performance, and FoF. Additionally, an intervention to improve balance confidence should be implemented in ODM with FoF.

## Figures and Tables

**Table 1:** Characteristics in groups based on the history of DM and performance on the mCTSIB.

Group	Number of participants	Age (year)	Duration since DM diagnosis (year)	FBS (mg/dL)	HbA1C (%)
ONDM	20	67.6 ± 5.2	—	94.5 ± 16.0	5.7 ± 0.6
ODM-0	28	66.0 ± 5.6	11.6 ± 5.9	130.3 ± 31.5^a^	7.7 ± 1.35^a^
ODM-1	44	68.8 ± 5.7	10.6 ± 6.3	151.7 ± 55.05^a^	7.6 ± 1.55^a^
Failed mCTSIB 3	1				
Failed mCTSIB 4	43				
ODM-2	20	71.3 ± 7.1^a,b^	9.6 ± 3.9	149.1 ± 55.25^a^	7.8 ± 1.75^a^
Failed mCTSIB 2 & 4	4				
Failed mCTSIB 3 & 4	16				
ODM-3	18	71.1 ± 9.0^a,b^	14.2 ± 7.2	149.2 ± 54.65^a^	7.7 ± 1.85^a^
Failed mCTSIB 1, 3 & 4	1				
Failed mCTSIB 1, 2, 3, & 4	3				
Failed mCTSIB 2, 3, & 4	14				

ONDM = older adults without diabetes; ODM-0 = older adults with diabetes without failed performance on mCTSIB; ODM-1 = older adults with diabetes who failed on one condition of mCTSIB; ODM-2 = older adults with diabetes who failed on two conditions of mCTSIB; ODM-3 = older adults with diabetes who failed on more than two conditions of mCTSIB; FBS = fasting blood sugar; HbA1C = hemoglobin A1C. ^a^Significantly different than ONDM (*p* < 0.05). ^b^Significantly different than ODM-0 (*p* < 0.05).

**Table 2:** Mean ± standard deviation of lower extremity muscle torque, fear of falling, mCTSIB, and timed up and go test.

	ONDM	ODM-0	ODM-1	ODM-2	ODM-3
	(*n* = 20)	(*n* = 28)	(*n* = 44)	(*n* = 20)	(*n* = 18)
Lower extremity muscle torque (Nm)					
Knee extensors	22.87 ± 1.86	19.72 ± 0.83	16.28 ± 0.76^a^	13.66 ± 0.98^a,b^	14.38 ± 1.08^a,b^
Knee flexors	17.64 ± 1.56	12.90 ± 0.51^a^	10.90 ± 0.50^a^	10.19 ± 0.74^a^	9.16 ± 0.58^a,b^
Ankle plantar flexors	25.53 ± 2.06	19.14 ± 0.69^a^	16.44 ± 0.63^a^	14.92 ± 1.29^a^	14.31 ± 0.83^a,b^
Ankle dorsiflexors	15.13 ± 1.50	13.42 ± 0.58	11.43 ± 0.48^a^	10.15 ± 0.66^a,b^	10.97 ± 0.52^a^
Total	81.17 ± 6.60	65.17 ± 2.13^a^	53.79 ± 2.35^a^	48.91 ± 3.36^a,b^	48.82 ± 2.31^a,b^
Fear of falling					
Total^#^	56.93 ± 6.14	66.84 ± 4.68	73.63 ± 3.74	75.71 ± 5.60	80.88 ± 5.89
Physical domain^#^	23.90 ± 3.08	28.78 ± 2.34	29.50 ± 1.87	31.33 ± 2.80	34.43 ± 2.95
Environmental domain^#^	17.72 ± 2.33	19.42 ± 1.78	22.20 ± 1.42	22.03 ± 2.13	23.64 ± 2.24
Psychological domain^#^	15.31 ± 1.71	18.64 ± 1.30	22.94 ± 1.04^a^	22.35 ± 1.56^a^	22.82 ± 1.64^a^
mCTSIB-Tol^$^	118.53 ± 2.89	119.56 ± 2.14	100.81 ± 1.71^a,b^	81.41 ± 2.56^a,b,c^	53.46 ± 2.70^a,b,c,d^
Timed up and go (s)^$^	9.83 ± 1.18	11.84 ± 0.87	12.22 ± 0.70	13.53 ± 1.05	14.84 ± 1.10^a^

*n* = number of participants; ONDM = older adults without diabetes; ODM-0 = older adults with diabetes without failed performance on mCTSIB; ODM-1 = older adults with diabetes who failed on one condition of mCTSIB; ODM-2 = older adults with diabetes who failed on two conditions of mCTSIB; ODM-3 = older adults with diabetes who failed on more than two conditions of mCTSIB. ^#^Adjusted values based on the sum of lower extremity torque as covariate using ANCOVA. ^$^Adjusted values based on covariates of the sum of lower extremity torque and the psychological domain of fear of falling using ANCOVA. ^a^Significantly different than ONDM (*p* < 0.05). ^b^Significantly different than ODM-0 (*p* < 0.05). ^c^Significantly different than ODM-1 (*p* < 0.05). ^d^Significantly different than ODM-2 (*p* < 0.05).

## Data Availability

The data used to support the findings of this study are available from the corresponding author upon request.
